# STUDENT SERVICES ON CAMPUS: SUPPORTING OLDER STUDENTS

**DOI:** 10.1093/geroni/igae098.1434

**Published:** 2024-12-31

**Authors:** Natalie Galucia

**Affiliations:** Washington University in St. Louis, St. Louis, Missouri, United States

## Abstract

Universities are experiencing shifts in age distributions of students and have an increasing need to embrace age-diversity on their campuses. Two recent studies explore this shift in demographics as they focused on staff members in student services and collected data on their perspectives regarding age-diversity and inclusion on campus. The first study involved focus groups with staff in admissions and career services to explore the challenges, opportunities and strategies related to serving an age-diverse student body. Themes regarded both the benefits of age-diversity but also the challenges they face in attracting and supporting older students. A second study involved staff from Diversity, Equity and Inclusion offices at universities across the country. These DEI officers acknowledged age as an identity relevant to the student experience but they did not elevate age bias or discrimination as a priority for their work. In both studies, affinity groups for non-traditionally aged students were suggested as a strategy. Discussion will be included around the experiences with starting a student group, called Next Move, focused on providing resources and support for older students at the Brown School at WashU. Opportunities and challenges for creating support groups for these students will be discussed.

